# Morpho-Physiological and Hormonal Response of Winter Wheat Varieties to Drought Stress at Stem Elongation and Anthesis Stages

**DOI:** 10.3390/plants12030418

**Published:** 2023-01-17

**Authors:** Jurica Duvnjak, Ante Lončarić, Lidija Brkljačić, Dunja Šamec, Hrvoje Šarčević, Branka Salopek-Sondi, Valentina Španić

**Affiliations:** 1 Department for Breeding & Genetics of Small Cereal Crops, Agricultural Institute Osijek, Južno Predgrađe 17, 31000 Osijek, Croatia; 2 Faculty of Food Technology Osijek, University of J.J. Strossmayer in Osijek, Franje Kuhača 18, 31000 Osijek, Croatia; 3 Ruđer Bošković Institute, Bijenička Cesta 54, 10000 Zagreb, Croatia; 4 Department of Food Technology, University Center Koprivnica, University North, Trg dr. Žarka Dolinara 1, 48000 Koprivnica, Croatia; 5 Faculty of Agriculture, University of Zagreb, Svetošimunska Cesta 25, 10000 Zagreb, Croatia

**Keywords:** abscisic acid, drought stress, salicylic acid, winter wheat

## Abstract

Drought stress can significantly reduce wheat growth and development as well as grain yield. This study investigated morpho-physiological and hormonal (abscisic (ABA) and salicylic (SA) acids) responses of six winter wheat varieties during stem elongation and anthesis stage as well grain yield-related traits were measured after harvest. To examine drought response, plants were exposed to moderate non-lethal drought stress by withholding watering for 45 and 65% of the volumetric soil moisture content (VSMC) for 14 days at separate experiments for each of those two growth stages. During the stem elongation phase, ABA was increased, confirming the stress status of plants, and SA showed a tendency to increase, suggesting their role as stress hormones in the regulation of stress response, such as the increase in the number of leaves and tillers in drought stress conditions, and further keeping turgor pressure and osmotic adjustment in leaves. At the anthesis stage, heavier drought stress resulted in ABA accumulation in flag leaves that generated an integrated response of maturation, where ABA was not positively correlated with any of investigated traits. After harvest, the variety Bubnjar, followed by Pepeljuga and Anđelka, did not significantly decrease the number of grains per ear and 1000 kernel weight (except Anđelka) in drought treatments, thus, declaring them more tolerant to drought. On the other hand, Rujana, Fifi, and particularly Silvija experienced the highest reduction in grain yield-related traits, considering them drought-sensitive varieties.

## 1. Introduction

Wheat (*Triticum aestivum* L.) is one of three major cereal crops providing daily calories and protein intake with annual global production of 780 million tons [1]. It is expected that demand for wheat will increase by up to 60% by 2050, whereas wheat production might be decreased by 29% due to climate change imposed by environmental stress [2]. This may lead to an uncertain future for world resources due to an increase in global average surface temperature [3]. Thus, drought is an important and challenging issue in wheat research because it has become one of the major problems worldwide as a result of climate change [4] that negatively affects wheat growth, development, and grain yield [5].

Wheat plants may be more susceptible to drought at critical growth stages such as germination and seedling stages [6], tillering and stem elongation stages [7], and anthesis and grain filling stages [8]. According to previous research by Sarto et al. [8], droughts with different intensities that occurred during different growth stages of crops differently influenced grain yield. During the germination stage, drought stress resulted in the reduction of germination rate and percentage, leading to prolonged germination time [9,10]. As drought stress levels increased, there was a significant decrease in the root and shoot fresh weight of wheat seedlings [11]. Furthermore, leaf wilting reflected the effect of drought stress on plant leaves during drought treatment [12]. Moreover, plant growth was hampered due to the turgor loss of plant cells [13]. Previously, drought impact on the plant density in the initial phase, on the tiller number per plant in the tillering phase, and on the plant height in the stem elongation phase was reported [8]. As the cell elongation was disrupted, wheat’s growth and height were also reduced [14]. Drought can shorten the stem elongation phase and consequently reduce the dry weight of ears and the number of fertile florets at anthesis resulting in lower grain yields [15]. Although drought impeded wheat performance at all growth stages, it was more critical during flowering and grain-filling stages resulting in substantial grain yield losses [16]. Moreover, drought stress influenced the fullness of wheat grains during grain filling [17]. It was reported that drought in the pre-anthesis stages decreased grain number per unit area, while drought in the post-anthesis stage affected the grain weight [18].

Aside from morphological adjustments, wheat plants can adapt themselves to drought conditions by activation of different molecular, biochemical or physiological processes [19]. In that case, plants take a step at the cell level against drought stress through the development of a mechanism that maintains the osmotic adjustments at the tissue level [20]. In general, drought-tolerant plants accumulate soluble sugars, proline content, amino acids, chlorophyll content, and enzymatic and non-enzymatic antioxidant activities [21]. It was previously concluded that more drought-tolerant wheat varieties could conserve water content in photosynthetic tissue and are less affected by evapotranspiration water losses [22]. Further, phytohormones play a significant role in response to abiotic stress, besides their physiological functions and involvement in the regulation of most developmental functions in plants [23]. One of the most important signaling phytohormones under drought stress is abscisic acid (ABA) [24]. According to previous research, ABA was accumulated in the leaf apoplast and induced stomatal closure under drought stress, whereas stomatal closure prevented intracellular water loss [25]. Consequently, the photosynthesis of plants was also impaired by drought, with decreased carbon assimilation [26,27]. Further, ABA helped seeds overcome stress conditions and germinate only when conditions were suitable for germination [28]. Along with ABA, salicylic acid (SA) also has a major role in modulating the plant response to drought with diverse roles in physiological processes, including germination, flowering, photosynthesis, modulation of stomatal opening and closing, and thermotolerance [29,30]. It also regulates the deterioration of reactive oxygen species (ROS) and the function of the antioxidative system [31] and induces genes responsible for encoding chaperones, heat shock proteins, and secondary metabolites [32]. Protection against drought could be accomplished through the overproduction of SA via the enhanced activity of SA biosynthetic pathway enzymes [33]. In addition, signaling cross-talk of phytohormones, such as the interaction between SA and ABA, has been recorded under both normal and stressed conditions [34]. 

Our previous study reported the significant negative impact of osmotic stress caused by polyethylene glycol (PEG) treatments on germination and seedlings growth of six winter wheat varieties: Silvija, Rujana, Bubnjar, Fifi, Anđelka, and Pepeljuga [10]. The objectives of the present study were (i) to investigate the effects of drought application on morpho-physiological traits in six bread wheat varieties during stem elongation and anthesis stages, (ii) to investigate the impacts of two different intensities of droughts on winter wheat by studying the occurrence timing of droughts relative to the growth stage of winter wheat, (iii) to compare the performances of elite wheat varieties under drought in terms of ABA and SA concentrations, and (iv) to identify the superior wheat varieties that can be used in breeding for drought suffered environments. We hypothesize that morpho-physiological and hormonal stage-specific traits may be potential targets for the future selection of drought-tolerant wheat varieties and that drought-tolerant varieties in early developmental stages will manifest drought tolerance in the latter developmental stages.

## 2. Results

### 2.1. Stem Elongation Phase

**Figure 1:**
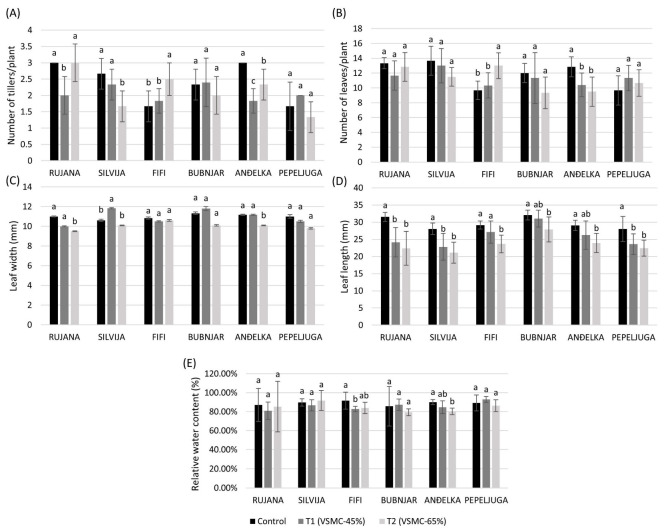
Number of tillers (**A**) and leaves (**B**) per plant, leaf length (**C**) and width (**D**), and relative water content (**E**) during the stem elongation stage in six winter wheat varieties. Data are average values of six biological replicates ± SD. Each biological replicate consisted of one plant. Different lowercase letters represent significantly different values (*p* < 0.05) within one variety under three treatments.

During the stem elongation stage, the number of tillers per plant was significantly reduced in Rujana by 50.0% and Anđelka by 63.6% at a 45% reduction of volumetric soil moisture content (VSMC), compared to the control (Figure 1A). At 65% reduction of VSMC, compared to the control, Silvija and Anđelka significantly reduced the number of tillers by 60.0 and 28.6%, respectively, while Fifi increased it by 33.3%. Varieties Rujana, Fifi, and Anđelka significantly increased the number of tillers at a 65% reduction of VSMC, compared to a 45% reduction of VSMC. 

The number of leaves per plant was considerably reduced at 45% and 65% reduction of VSMC, compared to the control, in Anđelka, while Fifi had a significantly higher number of leaves per plant at 65% reduction of VSMC, compared to the control (Figure 1B). 

Variety Silvija significantly increased leaf width at a 45% reduction of VSMC by 11.6%, compared to the control, while Rujana and Anđelka significantly reduced it by 13.6 and 9.5%, respectively, at a 65% reduction of VSMC. A significant reduction of leaf width was observed in the variety Silvia at a 65% reduction of VSMC, compared to a 45% reduction of VSMC (Figure 1C). At a 45% reduction of VSMC, a significant reduction of leaf length was observed in Rujana, Silvija, and Pepeljuga, compared to the control (Figure 1D). The leaf length was significantly reduced in all varieties at a 65% reduction of VSMC compared to the control. The greatest reduction was noticed in Rujana (29.0%) and Silvija (24.7%). 

Variety Fifi significantly reduced relative water content (RWC) by 8.5% at a 45% reduction of VSMC, compared to the control, while Anđelka significantly reduced it by 10.9% at a 65% reduction of VSMC, compared to the control (Figure 1E). 

**Figure 2:**
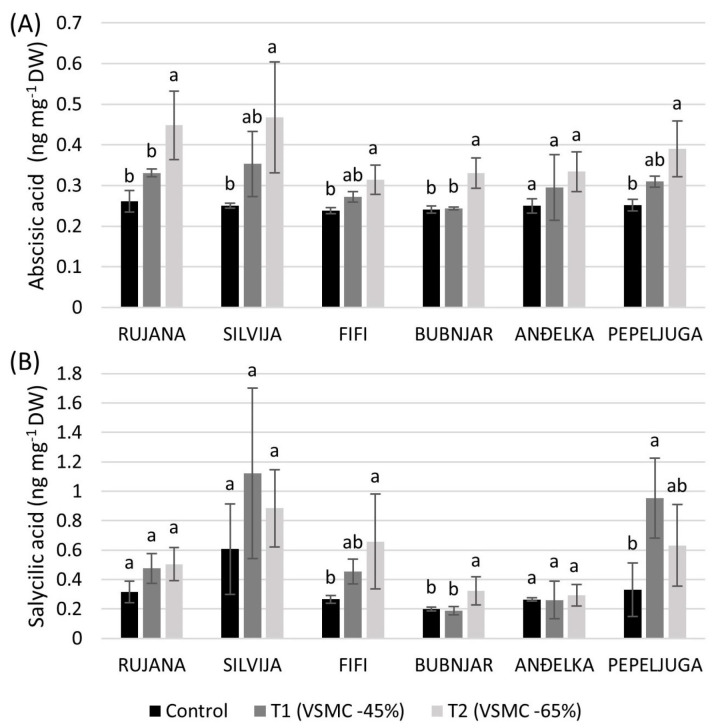
Concentration of abscisic acid (**A**) and salicylic acid (**B**) in leaves during the stem elongation stage of six winter wheat varieties. Data are average values of three biological replicates ± SD. Each biological replicate consisted of one plant. Different lowercase letters represent significantly different values (*p* < 0.05) within one variety under three treatments.

All varieties showed an increasing trend of ABA concentration in leaves with increasing drought stress, but statistically significant changes, compared to the control, were observed only at 65% reduction of VSMC in varieties Silvija (46.5%), Rujana (41.7%), Pepeljuga (35.7%), Bubnjar (27.1%), and Fifi (24.3%) (Figure 2A).

**Figure 3:**
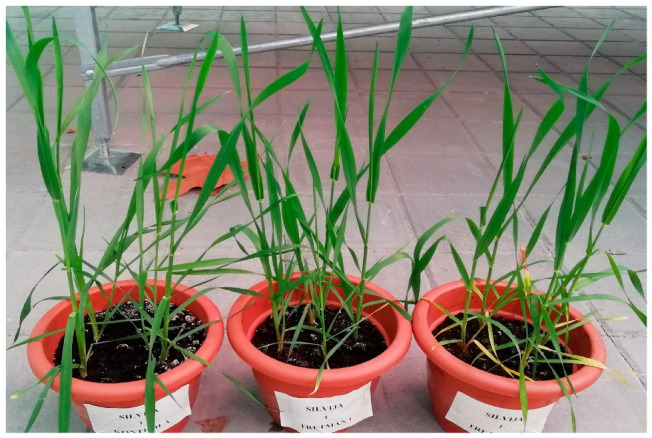
Withering of the bottom leaves at 65% reduction of volumetric soil moisture content (VSMC) in variety Silvija two weeks after drought treatments (pot with control plants, pot with plants at 45% reduction of VSMC, and pot with plants at 65% reduction of VSMC).

Bubnjar and Fifi significantly increased SA concentration in leaves at a 65% reduction of VSMC by 38.4 and 59.7%, respectively, compared to the control, while Pepeljuga significantly increased it by 65.4% at a 45% reduction of VSMC (Figure 2B). Signs of the withering of bottom leaves were observed at a 65% reduction of VSMC in some varieties (Figure 3).

### 2.2. Anthesis Stage

**Figure 4:**
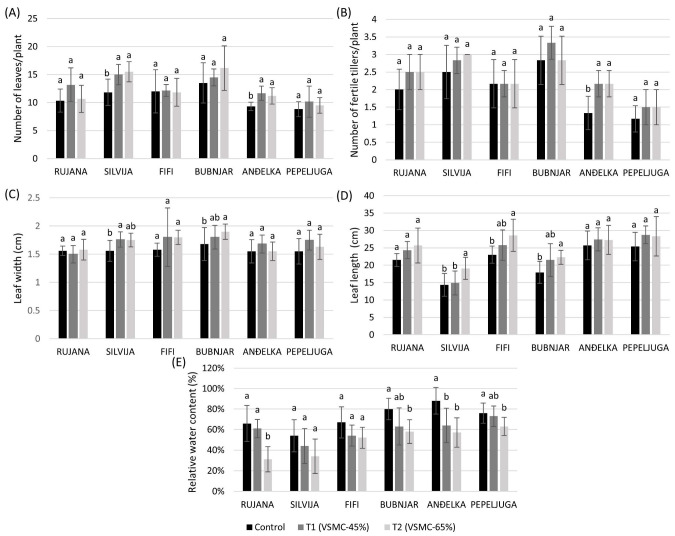
Number of leaves (**A**) and fertile tillers (**B**) per plant, leaf width (**C**) and length (**D**), and relative water content (**E**) during the anthesis stage of six winter wheat varieties. Data are average values of six biological replicates ± SD. Each biological replicate consisted of one plant. Different lowercase letters represent significantly different values (*p* < 0.05) within one variety under three treatments.

Only variety Anđelka significantly increased the number of leaves and fertile tillers per plant by 62.5 and 62.5%, and by 25.0 and 19.6%, respectively, at 45 and 65% reduction of VSMC, compared to the control, while Silvija significantly increased only the number of leaves per plant by 26.8 and 31.0% at the two drought intensities (Figure 4A,B). 

Variety Silvija significantly increased the leaf width by 12.8%, at a 45% reduction of VSMC, compared to the control, while Bubnjar significantly increased it by 13.1% at a 65% reduction of VSMC (Figure 4C). 

The leaf length was significantly increased by 33.3, 24.4, and 24.1% in Silvija, Bubnjar, and Fifi, respectively, at a 65% reduction of VSMC, compared to the control (Figure 4D). Significant reductions of RWC by 52.9, 35.2, 27.5, and 17.1% were observed in Rujana, Anđelka, Bubnjar, and Pepeljuga, respectively, at 65% reduction of VSMC, compared to the control, while for Anđelka the reduction of RWC was also observed at 45% reduction of VSMC (Figure 4E).

**Figure 5:**
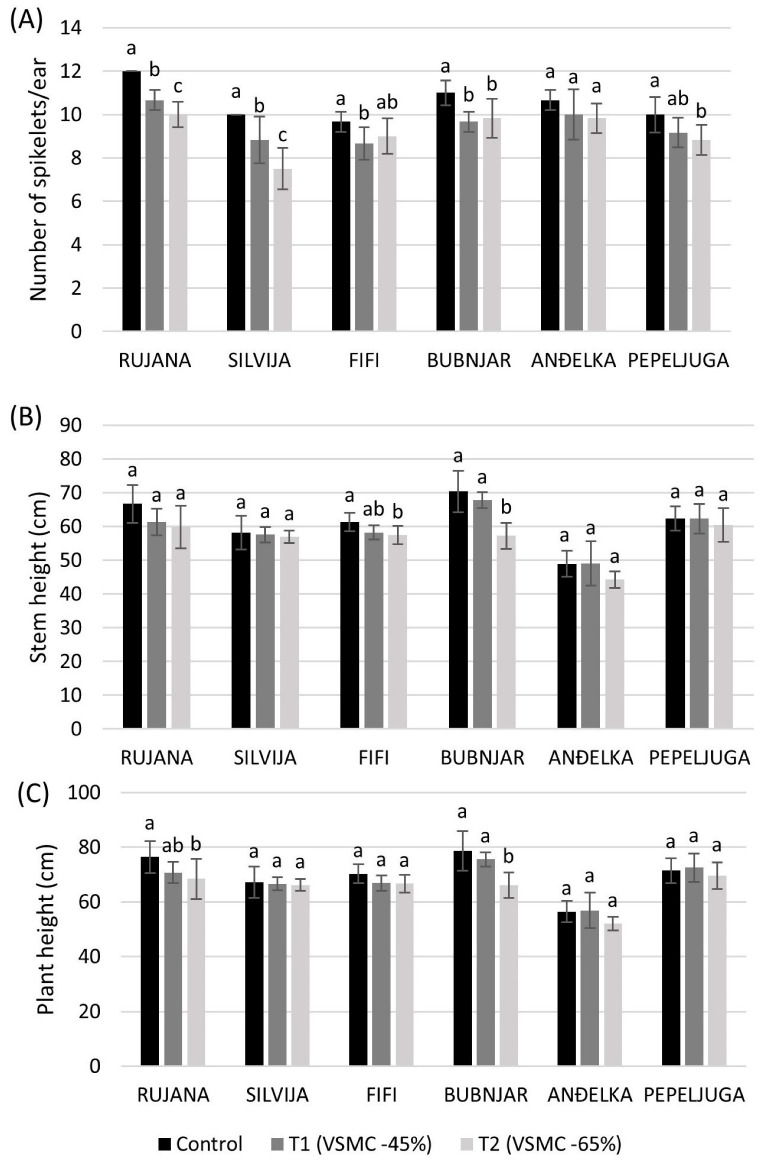
Number of spikelets per ear (**A**), stem height (**B**), and plant height (**C**). Data are average values of six biological replicates ± SD. Each biological replicate consisted of one plant. Different lowercase letters represent significantly different values (*p* < 0.05) within one variety under three treatments.

The number of spikelets per ear was significantly reduced by 12.1, 11.6, 11.1, and 10.3% in Bubnjar, Silvija, Rujana, and Fifi, respectively, and at a 45% reduction of VSMC, compared to the control, while varieties Silvija, Rujana, Pepeljuga, and Bubnjar significantly reduced it by 25.0, 16.6, 11.6, and 10.6%, respectively, at 65% reduction of VSMC, compared to the control (Figure 5A). In the control plants, the non-significant higher stem height was recorded for all varieties, compared to drought treatments, by 45 and 65% reduction of VSMC, but only varieties Fifi and Bubnjar significantly reduced it at 65% reduction of VSMC, compared to control plants, by 6.3 and 18.7%, respectively (Figure 5B). There was no significant change in plant height between plants in control and a 45% reduction of VSMC. A significant reduction of plant height was recorded at 65% reduction of VSMC, compared to control plants, for Rujana and Bubnjar by 10.5 and 16.0% (Figure 5C).

**Figure 6:**
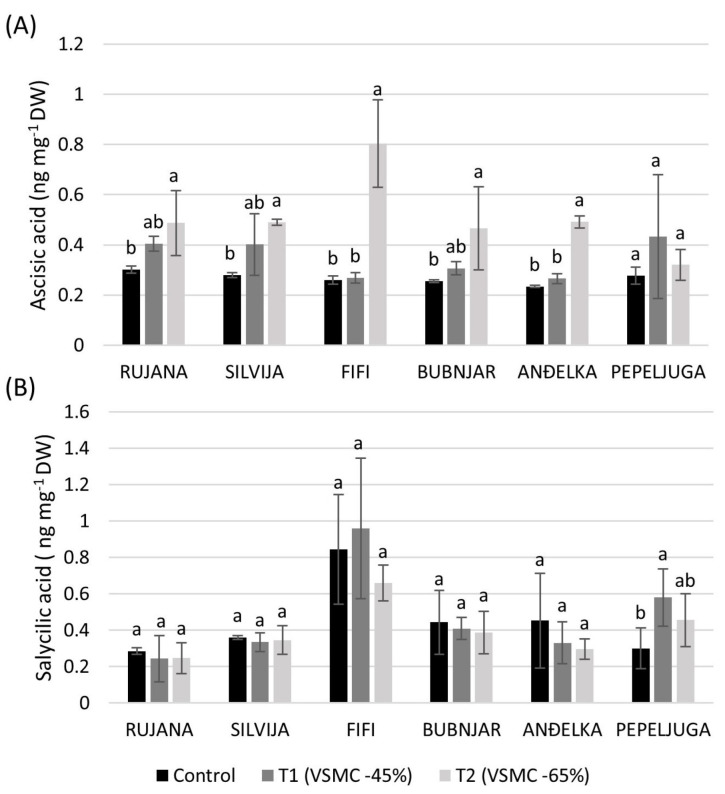
Concentration of abscisic acid (**A**) and salicylic (**B**) acid in flag leaves during the anthesis stage of six winter wheat varieties. Data are average values of three biological replicates ± SD. Each biological replicate consisted of one plant. Different lowercase letters represent significantly different values (*p* < 0.05) within one variety under three treatments.

Significant increase in the concentration of ABA occurred in flag leaves of Fifi, Anđelka, Bubnjar, Silvija, and Rujana at 65% reduction of VSMC, compared to control plants, by 67.7, 52.4, 45.2, 43.0, and 38.1%, respectively (Figure 6A). Only Pepeljuga significantly increased SA in flag leaves at a 45% reduction of VSMC, compared to the control, by 48.1% (Figure 6B). 

### 2.3. Grain Yield-Related Measurements after Harvest 

**Figure 7:**
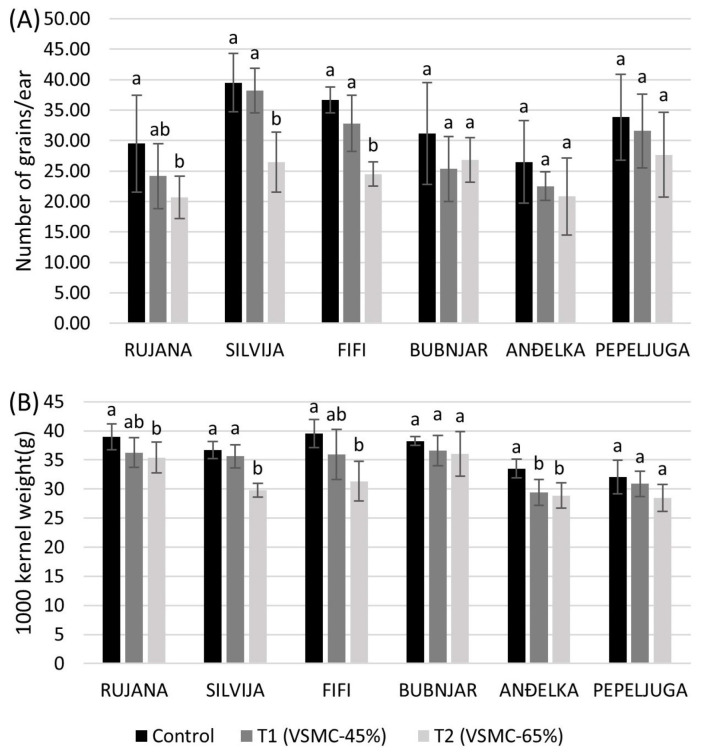
Number of grains per ear (**A**) and 1000 kernel weight (**B**) after harvest of six winter wheat varieties. Data are average values of six biological replicates ± SD. Each biological replicate consisted of one plant. Different lowercase letters represent significantly different values (*p* < 0.05) within one variety under three treatments.

A reduction of VSMC by 45% did not cause significant changes in the number of grains per ear in comparison to the control (Figure 7A). The number of grains per ear was significantly reduced by 33.2, 32.9, and 29.9% in Fifi, Silvija, and Rujana, respectively, at 65% reduction of VSMC, compared to the control, while no significant reduction was recorded in other varieties. Only Anđelka significantly reduced 1000 kernel weight at 45% reduction of VSMC, compared to the control, while significant reduction by 20.7, 18.7, 13.8, and 9.1% was observed in Fifi, Silvija, Anđelka, and Rujana, respectively, at 65% reduction of VSMC, compared to the control (Figure 7B).

### 2.4. Correlation and Principal Component Analysis 

**Figure 8:**
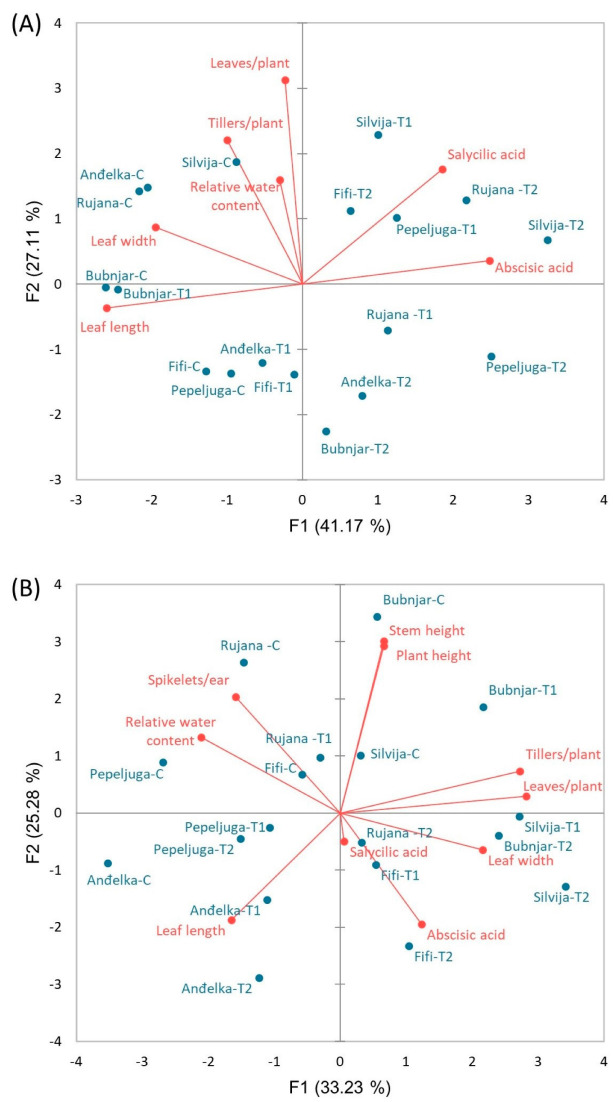
Principal component analysis (PCA) showing the relationship of morpho-physiological traits and stress hormones at (**A**) stem elongation stage and (**B**) anthesis stage (Rujana, Silvija, Fifi, Anđelka, Bubnjar, and Pepeljuga) under two drought regimes (T1 = VSMC-45% and T2 = VSMC-65%) and control (C). PCA was performed on the correlation matrix of average values of morpho-physiological attributes (number of leaves, leaf length, leaf width, number of tillers, RWC, number of spikelets per ear, stem height, plant height), and concentrations of stress hormones (abscisic acid and salicylic acid).

**Figure 9:**
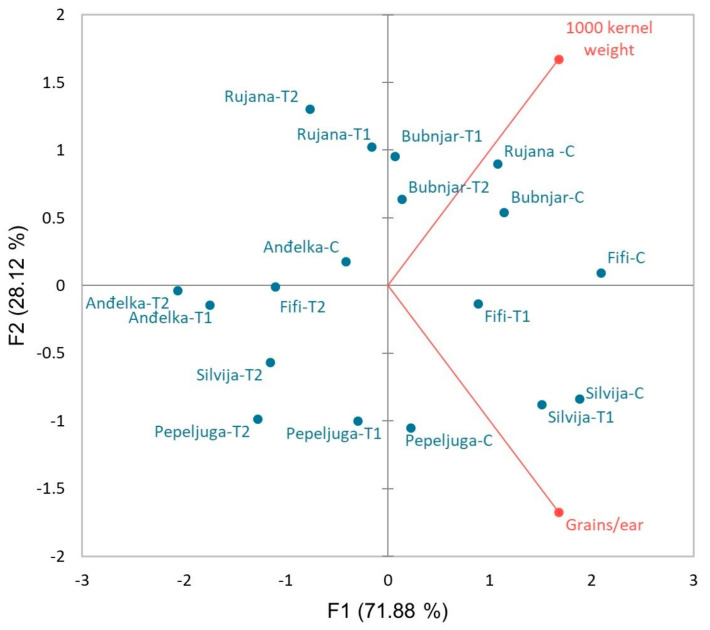
Principal component analysis (PCA) showing the relationship between two-grain yield-related traits after harvest for six winter wheat varieties (Rujana, Silvija, Fifi, Anđelka, Bubnjar, and Pepeljuga) under two drought regimes (T1 = VSMC-45% and T2 = VSMC-65%) and the control ©. PCA was performed on the correlation matrix of average values of grain yield-related traits (number of grains per ear, 1000 kernel weight).

Correlation analysis at the stem elongation stage (Table S1), anthesis stage (Table S2), and after harvest (Table S3) were performed to show relationships among traits. Further, to visualize the relationships between morpho-physiological traits, plant stress hormones, and the level of drought tolerance of six winter wheat varieties, PCA analysis was conducted for two developmental stages, stem elongation (Figure 8A) and anthesis stage (Figure 8B) as well as grain yield-related data obtained after harvest (Figure 9). 

At the stem elongation stage, it was shown that leaf length was in significant positive correlation with leaf width, as well as the number of leaves per plant with the number of tillers per plant. ABA and SA were in significant negative correlation with leaf length, as well as ABA with leaf width. In addition, ABA and SA were significantly positively correlated (Table S1). The PCA biplot showed that at the stem elongation stage, 41.17% of the total variability was explained by the first principal component (PC1) and 27.11% by the second principal component (PC2) (Figure 8A). The first two principal components (PCs) together explained 68.28% of the total variability. As can be seen from the biplot, morpho-physiological traits were grouped on the left side, while stress hormones were grouped on the right side of the PCA plot, indicating a negative correlation between morpho-physiological traits and stress hormone concentrations. At the same time, the positioning of wheat varieties in the control conditions and two treatments showed that the controls of all varieties were grouped closely on the left side of the PCA plot, while the treatments (T1 and T2) were shifted toward the right side of the PCA plot. The shift was larger the more drastic changes the varieties exhibited under stress conditions. Furthermore, the shifts to the right were consistent with the severity of the stress (the T2 treatments were positioned further to the right compared to the T1 treatments for all varieties). Accordingly, the variety Silvija experienced the most drastic changes under drought, while Bubnjar experienced the less drastic changes, indicating that Silvija was the most sensitive and Bubnjar the most tolerant variety at the stem elongation stage to the applied drought treatments.

At the anthesis stage, a significant positive correlation was observed between the number of leaves per plant and the number of fertile tillers per plant, the number of leaves per plant and the leaf width, the number of spikelets per ear and RWC, as well as between stem and plant height. The number of leaves per plant and leaf length, the number of fertile ears per plant and RWC, the number of fertile tillers per plant and leaf length, as well as the number of spikelets per ear and leaf width, were negatively correlated. A significant negative correlation was also observed between ABA and RWC, as well as between SA and the number of spikelets per ear. On the other hand, SA was in a significant positive correlation with leaf width (Table S2). PCA showed that PC1 accounted for 33.23% and PC2 for 25.28% of the total variability (Figure 8B), explaining together 58.51% of the total variability. As can be seen from the biplot, traits such as RWC, leaf length, and spikelets per ear were positioned opposite to stress hormones, stem and plant height, tillers per plant, leaves per plant, and leaf width. Varieties were positioned according to certain changes exhibited under stress treatments. Overall, Bubnjar, Pepeljuga, and Anđelka seem to undergo less drastic changes under stress compared to their controls, while Rujana, Fifi, and particularly Silvija experienced more drastic changes under stress conditions suggesting their level of drought tolerance at the anthesis stage.

At final, after harvest, no significant correlations were observed between traits (Table S3). 

PCA considering grain yield-related traits (grains per ear and 1000 kernel weight) that were measured after harvest (Figure 9) revealed that PC1 accounted for 71.88% and PC2 for 28.12% of the total variability explaining together 100.00% of the total variability. The angle between the vectors of grains per ear and 1000 kernel weight on the PCA biplot is close to 90°, which means that the correlation between the two traits is close to zero. Furthermore, based on grain yield related-traits, the relative position of the control and T1 and T2 treatment differs among varieties. As can be seen, there is a small shift in Bubnjar under drought treatments compared to the control, indicating its good performance under drought stress. Pepeljuga and Anđelka also showed a relatively small reduction in grain yield-related traits under stress conditions. On the other hand, Rujana, Fifi, and particularly Silvija showed a more drastic reduction in grain yield-related traits under stress conditions. In all varieties, except Bubnjar, the reduction in grain yield-related traits follows stress severity. 

## 3. Discussion

To adapt to drought stress, wheat plants have developed mechanisms that manifest themselves in morphological, physiological, developmental, and molecular changes. Under drought conditions, the plant produces ROS, while the antioxidant protective enzyme system, flavonoids, and secondary metabolites play a role in the protection of the plant by detoxifying ROS [35,36]. Aside from their role in irreversible DNA damage and cell death, ROS are important signaling molecules that regulate normal plant growth and responses to stress [37]. For example, ROS species are involved in the regulation of stomatal behavior [38], which is further controlled by ABA and SA [39,40].

In the present study, we investigated the mechanisms underlying the correlation of two endogenous levels of stress hormones, ABA and SA, and morpho-physiological traits at stem elongation and anthesis stages under two intensities of drought stress. In our previous research, morpho-physiological and biochemical responses of six winter wheat varieties (Silvija, Rujana, Bubnjar, Fifi, Anđelka, and Pepeljuga) to osmotic stress treatments caused by 10 and 20% PEG at germination and seedlings stage were examined [10]. Accordingly, all varieties significantly reduced germination energy at 20% PEG. The reduction of germination energy ranged from 6.6% for the variety Rujana to 17.0% for the variety Silvija. Seedling growth was also reduced for all varieties in a dose-dependent manner of applied PEG. The highest shoot length reduction was observed for the variety Silvija, followed by the variety Fifi while the smallest reduction was obtained for varieties Bubnjar, Pepeljuga, and Anđelka, compared to the control. Thus, results suggested Silvija as the most sensitive while Bubnjar is the most tolerant variety to osmotic stress at germination and early seedlings stage [10]. Herein, we proceeded with the research of stress response of the same varieties to two drought regimes at further developmental stages: stem elongation and anthesis. Finally, grain yield-related traits of six wheat varieties under drought conditions were measured after harvest.

### 3.1. Drought Response of Wheat Varieties at Stem Elongation Stage

The onset of stem elongation coincides with the transition from the vegetative to the reproductive stage when spikelet primordia are formed from leaf primordia when the apex meristem differentiates [41]. Therefore, this period is critical for spike development [42], where a significant reduction in the number of spikelets and, thus, the final number of grains per spike under stress can occur [43]. As a result, grain yield formation could be affected when 50% of grain yield potential based on the maximum number of floret primordia could be lost [44]. Further, at the beginning of stem elongation starts the highest water consumption by plants [8]. Drought stress during the stem elongation stage also reduced the elongation of the stem and cell expansion, which was related to changes in the metabolism of some hormones [45]. Previously it was reported that parallel with the increase in water deficit, there was a decrease in the RWC and water potential in the leaf [46]. RWC in leaves is, furthermore, a parameter reported to be significantly lower in plants under drought treatments compared to the control plants [47]. However, this is variety specific, and in the current study, RWC values were reduced under drought only in varieties Fifi and Anđelka. Unfortunately, drought during the stem elongation phase has been much less studied, although it is an important phase in the study of drought stress. 

In the current research, the number of tillers per plant was significantly reduced in varieties Silvija and Anđelka at a 65% reduction of VSMC, compared to the control, with a more pronounced reduction in variety Silvija. Rujana and Anđelka significantly reduced the number of tillers per plant already at a 45% reduction of VSMC, with a stronger reduction in tiller number of Anđelka under that treatment, compared to a 65% reduction of VSMC. In all varieties, leaf expansion (length and width) decreased significantly with increasing drought severity, compared to control, although differences were found among varieties. This is in agreement with the previous study, which reported genotypic variation in growth response to temperature for wheat leaf elongation rate [48]. In the current research, it is important to note that the reduction of leaf length was least pronounced for variety Bubnjar at 65% reduction of VSMC, compared to other varieties. Our results are in agreement with those of Qaseem et al. [49], who reported a reduction in tillering under drought conditions at the stem elongation stage. According to Urbanavičiūtė et al. [50], the decrease in the number of leaves and tillers under drought was variety specific, which is consistent with our results. In addition, Urbanavičiūtė et al. [50] concluded that varieties were more tolerant to drought due to their successful development of tillers under stress conditions. Tiller formation can be affected mainly by drought and nutrient deficiency [51,52]. Moreover, the number of tillers and leaves were significantly positively correlated in the current research, which is in agreement with the results of Miralles and Richards [53], suggesting that tiller and leaf growth were closely coordinated in wheat plants. 

ABA showed a tendency to increase in all varieties with increasing drought severity. A significant increase was recorded in all varieties, except in Anđelka, under more drastic drought conditions. As the increase in ABA is in accordance with the stress that wheat varieties experienced under drought treatments, the concentration of this hormone can serve as a good stress marker. ABA triggers stress signaling and tolerance in plants [54] and acts as an inhibitor of plant growth under water deficit [55]. Its concentration rapidly increases to initiate stomatal closure in the plant [56] and stimulate root cell elongation [57] but at the expense of the number of tillers and leaves. During drought stress, very rapid ABA-mediated closure of stomata will occur to limit water loss by evapotranspiration [25,58]. Furthermore, as the stomata close, the entry of CO_2_ into the mesophyll also decreases, with negative consequences for the net photosynthetic rate [59]. It was previously concluded that photosynthesis is one of the most sensitive processes to water deficit [60]. It has been previously reported that wheat plants accumulate inorganic solutes such as potassium, calcium, silicon, and SA in their cytosol to maintain cell turgor by lowering their osmotic potential under drought stress [61]. Our results showed that SA has a tendency to increase with drought stress, although the changes were not always statistically significant. Besides its role as a plant hormone and signaling molecule, SA is a phenolic acid and, therefore, has antioxidant activity. The increase in SA under drought conditions may participate in protection against water deficit at this developmental stage. It was previously reported that SA alters key plant functions, including water relations [62] and stomatal functioning [63]. We may speculate that the increase in SA under drought conditions may be involved in the tolerance mechanisms. Since variety Bubnjar did not show significant reductions in leaf width, number of tillers and leaves per plant, and relative water content under both drought treatments, compared to the controls, we may suggest it as relatively tolerant to drought stress during the stem elongation phase. Indeed, PCA analysis confirmed that variety Bubnjar exhibited the least changes under the drought treatments in comparison to the control, while variety Silvija underwent the most drastic changes indicating its sensitivity to drought, at the stem elongation stage.

### 3.2. Drought Response of Wheat Varieties at Anthesis Stage and Resulting Grain Yield-Related Data 

Plants were also subjected to two drought intensities during the anthesis stage, one of the terminal phases of wheat development. According to the study by Morgun et al. [46,64], differences in investigated traits between varieties were more contrasting when drought was applied at the anthesis, compared to the stem elongation stage. The flowering stage or anthesis begins after heading, and at this stage, the anthers release their pollen, after which grains are formed [65]. According to previous research, drought stress during grain filling is the most yield-damaging to wheat due to impaired grain development associated with imbalanced levels of growth hormones [66]. In the present study, a significant increase in the number of leaves per plant was observed in varieties Silvija and Anđelka under both drought treatments, compared to the controls, while in Anđelka, a significant increase in the number of fertile tillers per plant was also recorded. The number of fertile tillers depended on environmental conditions and the time of tiller formation [67].

The shape, size, senescence, and waxiness of leaves can also contribute to drought tolerance [68]. In the present study, leaf size (width and length) did not change or showed a tendency to increase under stress conditions. RWC was significantly decreased in leaves of all varieties except Silvija and Fifi, suggesting higher water loss under drought conditions. 

It has been previously reported that wheat height decreased due to drought stress [49]. In the current study, a significant decrease in plant height due to heavier drought stress (65% reduction of VSMC) was observed in two varieties (Rujana and Bubnjar). This can be explained by the fact that cell elongation was disrupted by drought, affecting wheat’s growth and height. The reduction in plant height caused by drought was about 7% at the grain-filling stage [69]. This is in accordance with the current study, where there was a reduction in plant height caused by drought by 10%, on average, for all varieties. Furthermore, drought-tolerant plants tend to maintain lower plant height and plant area index to reduce the moisture demand and prevent moisture loss due to transpiration [70]. Therefore, the variety Bubnjar seems to respond to heavier drought stress by the significantly shortened stem and plant height.

At the anthesis stage, all varieties showed an increase in ABA under drought stress with significant changes at more severe drought treatments (except Pepeljuga). Pepeljuga was the only variety that did not significantly change ABA concentration between treatments, but it also was the only variety that increased SA at a 45% reduction of VSMC. 

Stress hormones such as ABA and SA are among the main signaling molecules that orchestrate plant stress response. The correlation between ABA’s endogenous level and stress tolerance is not unambiguous in the plant kingdom. The endogenous level of this hormone oscillates according to its metabolism, plant species, and organ/tissues, as well as the duration and severity of the drought stress. Application of exogenous ABA under water stress accelerated the accumulation of osmolytes and improved the water status of plants that, resulted in higher grain weight in susceptible wheat varieties [71]. Correlations between endogenous ABA increase and plant tolerance are somehow controversial in literature and obviously depend on plant species and developmental stage. There were examples of positive correlations between ABA level and tolerance (sunflower and switchgrass) which suggested that constitutively high ABA levels in tolerant varieties confer a better ability to cope with an adverse water deficit [72]. On the other hand, some native species from the arid regions showed that the highest ABA levels were found in drought-sensitive *Poa ligularis*, while the lowest ABA levels were identified in the highly tolerant xerophytic species *Papostypa speciosa* [72]. Besides the ABA level, ABA sensitivity is also an important trait for plant survival. Experiments on Arabidopsis and wheat suggested that plants with a high drought tolerance showed a significantly higher ABA sensitivity than the sensitive lines [73,74]. Previously, it was demonstrated that the overexpressor rice line in the *OsSta2* gene (*Oryza sativa* Salt tolerance activation 2-Dominant) exhibited hypersensitivity to ABA and showed increased tolerance to drought and salt stress [75]. Our data demonstrated that ABA is a good stress marker in all wheat varieties under drought. SA is another well-known stress hormone, although the role of SA may be even more controversial than ABA under abiotic stress conditions since some investigators have reported an enhancement of drought tolerance by SA application, whereas others claimed a reduction in drought tolerance. Generally, the impact of SA in stress conditions was highly dependent on the concentration applied. Experiments with exogenous treatments showed that low concentrations of SA decreased oxidative stress and enhanced drought tolerance in maize, wheat, tomato, bean, etc. [30]. Furthermore, SA-accumulating mutants of *A. thaliana* (adr1, myb96-1d, siz1, acd6, and cpr5) exhibited stomatal closure and improved drought tolerance [30,34]. Moreover, it was reported that SA is able to improve the stability of photosynthetic apparatus [76]. Our results showed that all varieties at later developmental stages showed a slight increase or did not change the level of SA or under drought conditions significantly. Since SA is phenolic acid with reported antioxidant activity, its presence in the plant may also be positive as a ROS scavenger. It is necessary to keep in mind that tolerance is the result of a complex network of action and cross-talk of different plant hormones in which ABA and SA play an important role.

PCA analysis summarized all changes that six varieties exhibited under two stress regimes. Accordingly, it may be concluded that Bubnjar is the most tolerant variety while Silvija is the most sensitive under applied drought stress treatments. 

It has been reported that the period of seven to ten days before anthesis and five days after anthesis is the most critical period for reproductive development [77]. The most damaging to the grain size of wheat was drought stress at and just after the anthesis stage [42], which coincides with the period of a drought treatment applied in the current study. Moreover, drought during the anthesis stage mainly caused a reduction in grain size [78]. All varieties showed a tendency to reduce the number of grains per ear as well as 1000 kernel weight under stress conditions, although the changes were not statistically significant in all cases. For example, varieties Bubnjar, Anđelka, and Pepeljuga did not significantly change the number of grains per ear compared to the controls. Previously it was reported that under drought stress, the average kernel weight was significantly reduced in all tested varieties compared to their controls [78]. This is partially in agreement with the current research where 1000 kernel weight was significantly reduced in four out of six tested wheat varieties, but a slight non-significant reduction was observed in all varieties at 65% reduction of VSMC, compared to the control. Only Anđelka significantly reduced grain yield-related traits at a 45% reduction of VSMC. Khalili et al. [79] reported that increased drought intensity significantly decreased the grain yield and harvest index of maize, which is in agreement with our results. During reproductive development, drought stress reduced the grain number in the ear of wheat [80], where premature abortion of flowers occurred, which resulted in a reduced number of potential grains in the ear [81]. In previous studies, the number of grains per ear and the weight of grains was also affected by the environment, including drought [82]. Drought occurrence during anthesis affected the number of grains per spikelet and the total number of grains per spike [42], which was also demonstrated in the current research.

Correlation of grain yield-related traits and varieties under stress conditions presented by PCA clearly showed that Bubnjar is the most drought-tolerant variety, followed by Pepeljuga and Anđelka, while Rujana, Fifi, and particularly Silvija, are more sensitive to drought.

## 4. Materials and Methods

### 4.1. Plant Material

Six winter wheat varieties (Rujana, Silvija, Fifi, Bubnjar, Anđelka, and Pepeljuga) of the Agricultural Institute Osijek were examined under drought conditions. Rujana is a taller variety and is later in maturity than the other studied varieties. Silvija is a variety with a longer vegetation period and has a tolerance to low temperatures. Fifi is a medium-early variety with higher grain protein content. Bubnjar is a medium-early variety that was previously characterized by better germination under drought stress [10]. Anđelka, a medium-early variety, is characterized by good tolerance to lodging and lower temperatures, while Pepeljuga is a medium-early variety with medium height.

### 4.2. Chemicals

Salicylic acid (SA) and *(+)-cis, trans* abscisic acid (ABA) were purchased from Fluka and Duchefa-Biochemie, respectively. The internal isotope labeled standard SA-d_6_ was purchased from Sigma-Aldrich, *(+)-cis, trans* ABA-d_6_ from Trc. MiliQ^®^ water (18.2 MΩcm^−1^; purified by MiliQ water purification system (Millipore, Bedford, MA, USA)) and HPLC gradient-grade methanol (J.T. Baker) were used with analytical-grade formic acid (FA) (Acros Organics) for mobile phase preparation. Acetic acid (AcOH) for extraction was purchased from Sigma-Aldrich.

### 4.3. Drought Stress during Two Growth Stages

After germination in distilled water, five-day-old wheat seedlings of each wheat variety were placed in a plant growth chamber to undergo a period of vernalization under conditions of 12 h day/12 h night (4/3 °C) for a period of six weeks. After that, two separate experiments were set up in a greenhouse (Gis Impro d.o.o., Vrbovec, Croatia) where each experiment included three treatments: (1) two intensities of drought during stem elongation stage (GS31) [66] and controlled treatment with regular irrigation, (2) two intensities of drought during anthesis stage (GS61) and controlled treatment with regular irrigation. Within each treatment, varieties were randomized according to the random block design in six replicates, each containing 4 plants/2.5 L pot filled with soil (pH-H_2_O: 5.5–7.0, organic matter: 70.0–85.0%, N (1/2 vol.): 100–200 mg L^−1^, P_2_O_5_ (1/2 vol.): 100–150 mg L^−1^, K_2_O (1/2 vol.): 200–400 mg L^−1^). Nitrogen (N) fertilization was carried out at the two-leaf development stage (GS12) using calcium ammonium nitrate (CAN) (27%N) of grain/plant and two protections against diseases and pests. The first one was carried out with the fungicide Falcon Forte (*spiroxamine* 224 g L^−1^, *tebuconazole* 148 g L^−1^, *prothioconazole* 53 g L^−1^) at the stem elongation stage (GS30), and the second one a week later with the insecticide Vantex (*gamma-cyhalothrin* 60 g L^−1^) after the emergence of aphids (GS31). During tillering, stage temperatures were maintained during the night at 8–12 °C (14 h) and daytime temperatures at 10–14 °C (10 h) with the maximum light intensity of 250 µmol m^−2^ s^−1^. When the stem started to elongate, the length of day and night were adjusted to become equal, and daytime temperatures were maintained at 15–18 °C, while night temperatures were set up at 11–14 °C. Before anthesis, the length of the day was increased to 14 h, and the temperatures were maintained at 21–24°C and night temperatures at 17–20 °C with the maximum light intensity of 750 µmol m^−2^ s^−1^. During stem elongation and anthesis stages, in two separate experiments, plants were subjected to different intensities of drought by reducing water content by 45% (T1) and 65% (T2) of the volumetric soil moisture content (VSMC) for two weeks. In both treatments, non-lethal, moderate drought stress was produced. In the controlled treatment, the VSMC was maintained at 30–35%, where along with other treatments, VSMC was measured daily by a soil moisture measuring device (TDR 150 Soil Moisture Meter, Spectrum Technologies, Aurora, CO, USA).

### 4.4. Morphological Traits and Relative Water Content (RWC)

During the stem elongation stage, the number of leaves and tillers was counted, while leaf length and width (mm) were measured by the ruler on the 14th day of the experiment, in six replicates.

During the anthesis stage on the 14th day of the experiment, leaf length and width (mm), stem height (mm), and plant height (mm) were measured by the ruler in six replicates, while the tiller and leaf number were counted. Stem and plant height were measured from the ground to the base and to the top of the ear, respectively. After maturity, six wheat ears were collected from each treatment for further analysis of seed morphology using a MARViN seed analyzer (MARViTECH GmbH, Wittenburg, Germany), where 1000 kernel weight and the number of seeds per ear were measured.

For relative water content (RWC) measurement, leaf samples were collected from control and drought treatments after 14 days in drought-treated plants during stem elongation and anthesis stage (10 × 10 mm diameter of the leaf/flag leaf) in six replicates. Leaf discs were weighed (FW) and immersed for 24 h in deionized water, after which the turgid weight (TW) was recorded. After 24 h of drying in a dryer at 105 °C, the dry biomass (DW) was recorded [83]. For RWC calculation, the following formula was used [84]:

$$RWC(\%)=\frac{FW-DW}{TW-DW}\times 100$$

### 4.5. Stress Hormone Analysis: ABA and SA

#### 4.5.1. Sample Preparation

After plant tissue sampling, the samples were frozen in liquid nitrogen and lyophilized in three replicates from the stem elongation and anthesis stages. Further, lyophilized samples were shredded by mortar and pestle in liquid nitrogen. 30 mg of powdered sample were extracted in 1 mL extraction solution (10% MeOH and 1% acetic acid containing 38.5 ng mL^−1^ of each internal isotope labeled standards SA-d_6_ and ABA-d_6_). After vortexing, the samples were placed in a Mixer Mill (Roche) (2 min, frequency 30,000 RPM), after which they were homogenized for 1 h at 4 °C. The samples were then centrifuged (10 min, 13,000 RPM), and 100 µL of clear solution was used for liquid chromatography with tandem mass spectrometry (LC-MS/MS) analysis of stress hormones.

#### 4.5.2. Preparation of Standard and Calibrant Solutions

Stock solutions of each analyte, including internal labeled standards, were prepared as 1 mg mL^−1^ solutions in methanol. Stock solutions were diluted together in 10% MeOH + 0.1% AcOH to yield a working solution of 1 μg mL^−1^ and 100 ng mL^−1^ of each substance. 100 ng mL^−1^ solutions of ABA and SA in 10% MeOH + 0.1% AcOH was used as a QC sample. In the QC sample, a mixture of isotope-labeled standards ABA-d_6_ and SA-d_6_ to a final concentration of 38.5 ng mL^−1^ was also added. All standard solutions and QC samples were stored at −20 °C.

The calibration samples were prepared from stock solutions of each analyte in 10% MeOH + 0.1% AcOH with the addition of internal standard solution (40 μL of spike mixture solution ABA-d_6_ and SA-d_6_ 1 µg mL^−1^, final concentration 38.5 ng mL^−1^). Particular calibration points were as follows: calibrant 1 ABA and SA 9.6 ng mL^−1^, calibrant 2 ABA and SA 24 ng/mL, calibrant 3 ABA and SA 48 ng mL^−1^, calibrant 4 ABA and SA 96 ng mL^−1^, calibrant 5 ABA and SA 192 ng mL^−1^ and calibrant 6 ABA and SA 480 ng mL^−1^, respectively. 5 µL of each calibrant was injected into the LC column. The calibration curve was obtained by linear regression; the peak area ratio (analyte/internal standard) was plotted versus the analyte concentration. Least-squares linear regression gave Spearman correlation coefficients of r^2^ = 0.9989 for ABA/ABA-d_6_ (regression lines y = 0.0223 + 0.0783) and r^2^ = 0.9969 for SA/SA-d_6_ (regression lines y = 0.359 − 1.5305). Quantification was performed by adding the unknown area in the calibration curve plotted peak area ratio (analyte/internal standard) versus analyte concentration.

QC sample and instrumental blank were injected after every few runs. During analysis, all instrumental blank samples were negative, and the area of each analyte in the QC samples was repeatable.

#### 4.5.3. LC-MS/MS Conditions

LC–MS/MS analysis was carried out using an Agilent Technologies 1200 series HPLC system equipped with a binary pump, a vacuum membrane degasser, an automated autosampler, and an injector interfaced with 6420 triple quadrupole mass spectrometer with electrospray ionization source (ESI) (Agilent Technologies Inc., Palo Alto, CA, USA).

The separation was performed on the Zorbax XDP C18 column (75 × 4.6 mm, 3.5 μm particle size) (Agilent Technologies Inc., Palo Alto, CA, USA). Solvents for the analysis were 0.1% formic acid (FA) in water (solvent A) and methanol (solvent B). The gradient was applied as follows: 0 min 50% A, 5–15 min 50% A–0% A, 15–17 min 0% A, 17.1–22 min 60% A. Flow rate was 0.3 mL min^−1^.

The electrospray ionization source was operated in negative mode, and samples were detected in the multiple reaction monitoring (MRM) modes with a dwell time of 10 ms per MRM transition. The desolvation gas temperature was 350 °C with a flow rate of 6.0 L min^−1^. The capillary voltage was 3.5 kV. The collision gas was nitrogen. The MRM transitions of precursor to product ion pairs were *m/z* 263–153 for ABA (quantifying ion), *m/z* 263–219 for ABA (qualifying ion), *m/z* 137–93 for SA, *m/z* 269–159 for ABA-d_6_ and *m/z* 141–97 for SA-d_6_ respectively. Fragmentor voltages were 100 V for ABA and ABA-d_6_ and 70 V for SA and SA-d_6_. The collision energy was set to 15 V for SA, 12 V for SA-d_6_, 3 V for ABA quantifying and ABA-d_6_, and 2 V for ABA qualifying transition.

All data acquisition and processing was performed using Agilent MassHunter software. ABA and SA concentrations were calculated and expressed as ng mg^−1^ DW.

### 4.6. Statistical Analysis

A randomized complete block design was applied both in the plant growth chamber and greenhouse to minimize the effect on the environment. Samples were collected from each pot, whereas morpho-physiological measurements were done in six biological replicates. Collected data were statistically analyzed using the Statistica software (version 14). Fisher’s LSD test at a 5% probability level was used to test differences among mean values. The results of analyzed morpho-physiological parameters were expressed as the mean value of six replicates ± standard deviation (SD). ABA and SA concentration was measured in three replicates of leaves and flag leaves at stem elongation and anthesis stage, respectively, and expressed as the mean value of three replicates ± standard deviation (SD). Correlation analyses were done by Spearman coefficient at *p* < 0.05 and *p* < 0.001. Principal component analysis was performed using Addinsoft XLSTAT (New York, NY, USA).

## 5. Conclusions

It was observed that certain morpho-physiological and hormonal changes were observed during drought stress, depending on varieties and stress intensity. ABA was increased in all six winter wheat varieties under drought, confirming the stress status of the plants. SA was increased at the stem elongation stage, while it did not change at the later developmental stage (anthesis stage). Correlation analysis and PCA showed that the variety Bubnjar, followed by Anđelka and Pepeljuga, experienced the least changes in morpho-physiological traits under stress conditions resulting in good grain yield-related traits after harvest. On the other hand, Fifi, Rujana, and particularly Silvija were more sensitive to drought and underwent more drastic changes in morpho-physiological traits at the stem elongation and anthesis stages, resulting in a greater reduction in grain yield-related traits. These results are in agreement with our previous study investigating the response of the same varieties to drought at germination and young seedlings stages. Overall, our findings indicated that wheat varieties differ in their ability to produce ABA under drought during all growth stages, whereas tolerance to drought is variety specific but remains the same for all developmental stages. Understanding the responses of different wheat to drought stress can help breeders to develop genetically improved drought-tolerant varieties.

## Data Availability

The data presented in this study are available in the article or supplementary material. The raw MS files are available on request from the corresponding author.

## Supplementary Materials

The following supporting information can be downloaded at: https://www.mdpi.com/article/10.3390/plants12030418/s1, Table S1: Correlation analysis of seven investigated traits in three treatments during the stem elongation stage; Table S2: Correlation analysis of ten investigated traits in three treatments during the anthesis stage; Table S3: Correlation analysis of two investigated traits in three treatments after harvest.
