# Supplementary material for: Morpho-Physiological and Hormonal Response of Winter Wheat Varieties to Drought Stress at Stem Elongation and Anthesis Stages
Source: Plants (Basel). 2023 Jan 17;12(3):418. doi: 10.3390/plants12030418 (PMC9921141; doi:10.3390/plants12030418)
Supplement: Supplementary file 1 [file plants-12-00418-s001.zip › plants-2140979-supplementary.pdf]

Table S1. Correlation analysis of seven investigated traits in three treatments during stem elongation stage

	Leaf length	Leaf width	Number of leaves/ plant	Number of tillers/ plant	Relative water content	Abscisic acid	Salicylic acid
Leaf length	1	0.60**	0.01	0.26	0.13	-0.91**	-0.77**
Leaf width		1	0.24	0.23	0.32	-0.65**	-0.42
Number of leaves/ plant			1	0.68**	0.24	-0.002	0.31
Number of tillers/ plant				1	-0.06	-0.16	-0.14
Relative water content					1	-0.35	0.12
Abscisic Acid						1	0.67**
Salicylic acid							1

**-significant at 0.01

Table S2. Correlation analysis of ten investigated traits in three treatments during anthesis stage

	Leaves/ plant	Fertile tillers/ plant	Spikelets / ear	Leaf width	Leaf length	Stem height	Plant height	Relative water content	Abscisic acid	Salicylic acid
Leaves/ plant	1.00	0.90**	-0.30	0.58*	-0.57*	-0.05	-0.09	-0.45	0.20	0.05
Fertile tillers/ plant		1.00	-0.16	0.46	-0.65**	0.01	-0.01	-0.52*	0.27	-0.16
Spikelets/ear			1.00	-0.62**	-0.21	0.26	0.28	0.51*	-0.40	-0.51*
Leaf width				1.00	0.00	-0.03	-0.06	-0.33	0.23	0.52*
Leaf length					1.00	-0.23	-0.22	0.14	0.20	0.27
Stem height						1.00	0.99**	0.40	-0.27	0.02
Plant height							1.00	0.39	-0.24	0.02
Relative water content								1.00	-0.68**	0.20
Abscisic acid									1.00	-0.27
Salicylic acid										1.00

**-significant at 0.01; *-significant at 0.05

Table S3. Correlation analysis of two investigated traits in three treatments after harvest

	Number of grains/ear	1000 kernels weight
Number of grains/ ear	1.00	0.42
1000 kernels weight		1.00
